# Assessment of the Antitumor Potential of Umbelliprenin, a Naturally Occurring Sesquiterpene Coumarin

**DOI:** 10.3390/biomedicines8050126

**Published:** 2020-05-18

**Authors:** Iram Shahzadi, Zain Ali, Seung Ho Baek, Bushra Mirza, Kwang Seok Ahn

**Affiliations:** 1 Plant Molecular Biology Lab, Institute of Biological Sciences, Department of Biochemistry, Quaid i Azam University, Islamabad 45320, Pakistan; iramshahzadi@bs.qau.edu.pk; 2 Molecular Cancer Therapeutics Lab, Institute of Biological Sciences, Department of Biochemistry, Quaid i Azam University, Islamabad 45320, Pakistan; zainali@bs.qau.edu.pk; 3 College of Korean Medicine, Dongguk University, 32 Dongguk-ro, Ilsandong-gu, Goyang-si, Gyeonggi-do 10326, Korea; baekone99@gmail.com; 4 Department of Science in Korean Medicine, College of Korean Medicine, Kyung Hee University, 24 Kyungheedae-ro, Dongdaemun-gu, Seoul 02447, Korea

**Keywords:** umbelliprenin, sesquiterpene, signaling cascades, apoptosis, Wnt, NF-ĸb

## Abstract

Cancer is one of the greatest causes of mortality worldwide. The prevalence rates of different types of cancer is increasing around the world as well. Limitations in chemotherapy and radiotherapy, owing to multiple side effects including cytotoxic effects of antitumor compounds on normal cells as well as the development of resistance to these treatment options in patients, create a serious threat to successful treatment of cancer. The use of natural compounds to prevent and treat cancers has been found to be quite effective, with fewer adverse effects found in patients. Umbelliprenin (UMB) is a naturally occurring sesquiterpene compound found in *Ferula* species and recently in *Artemisia absinthium*. Many studies have highlighted the antitumor potential of UMB in different cancer cell lines as well as in animal models. UMB exerts its anticancer actions by regulating extrinsic and intrinsic apoptotic pathways; causing inhibition of the cell cycle at the G0/G1 phase; and attenuating migration and invasion by modulating the Wnt signaling, NF-ĸB, TGFβ, and Fox3 signaling pathways. UMB also affects the key hallmarks of tumor cells by attenuating tumor growth, angiogenesis, and metastasis. This review provides an insight into the role of UMB as a potential antitumor drug for different malignancies and highlights the signaling cascades affected by UMB treatment in diverse tumor cell lines and preclinical models.

## 1. Introduction

Cancer is the greatest cause of mortality in both developed and developing countries. Cancer is defined by uncontrolled cell division and can also metastasize to other parts of the body. Normal cells can distinguish between unusual malignant and normal cell growth with the help of tumor-suppressing mechanisms present in the cell. The issue, however, emerges when various environmental influences (such as carcinogens, radiation, infections, and so forth) limit the function of these tumor-suppressing genes [1,2,3,4,5,6]. According to a recent report, the probability of being diagnosed with an aggressive form of cancer is anticipated to soon be as high as 40%. In 2018, approximately 18.1 million new cancer cases (17.0 million excluding non-melanoma skin cancer) and 9.6 million cancer deaths were reported (9.5 million excluding non-melanoma skin cancers). Globally, lung cancer is the most frequently diagnosed cancer (11.6% of total cases), followed closely by breast cancer and prostate cancer (7.1%), and colorectal cancer (6.1%). In addition, lung cancer is also responsible for the most cancer deaths (18.4% of all cancer deaths), followed by colorectal cancer (9.2%), stomach cancer (8.2%), and hepatic cancer (8.2%) [7]. Cancer mortality can be reduced by understanding the etiology of cancer, through the advancement of prevention strategies and diagnostic techniques, and through the discovery of novel treatment modalities [8]. While cancer therapies include radiotherapy, surgery, chemotherapy, and targeted molecular therapy have stabilized the prevalence of cancer to some extent [9]. The conventional therapeutic approaches have unfortunately been found to be less effective than desired because of numerous factors, including significant toxicity in addition to the capacity of cancer cells to develop resistance to available treatments [10].

There has been a shift in recent years towards the frequent use of natural substances and herbal medicines for cancer treatment, as they are environmentally more sustainable and lack major side effects [11,12,13,14,15,16]. The first recorded use of natural products as medicinal products dates back to 2600 BC in Mesopotamia. More than 700 drugs were also recorded in the “Ebers Papyrus” in 1550 BC [17,18]. Additionally, traditional Chinese medicine (TCM) has been well known for thousands of years [19,20]. In addition, the Indian Ayurveda system has been applied to cure various ailments since the first millennium BC [21]. Natural-product-based drug development has been also linked with some difficulties, such as availability, detection of bioactive compounds, difficulty of collecting wild specimens, etc. [22]. Therefore, it is a difficult task to assess the exact molecular mechanisms of action of natural products [23]. In terms of drug development and study, various natural phytochemicals that can be used to treat various diseases, including cancer, are becoming important [24,25,26,27,28,29]. Various studies have shown that phytochemicals can be effective against cancers of different types [30,31,32,33,34]. They demonstrate substantial efficacy via alterations in the initiation, growth, and progression of cancer, and by interrupting multiple mechanisms such as differentiation, cell proliferation, angiogenesis, apoptosis, and metastasis [35,36,37,38,39]. The limited efficacy and severe side effects of traditional chemotherapeutic agents have prompted researchers to attempt to discover natural antitumor compounds with fewer side effects.

Prenyloxycoumarins are a group of secondary metabolites found primarily in plants of the Rutaceae and Umbelliferae families [40]. Auraptene, umbelliprenin, and 7-isopentyloxycoumarin are the most extensively studied prenylated coumarins. These compounds display pleiotropic biological and pharmacological activities, which have drawn researchers’ attention in the past two decades [41].

## 2. Chemistry of Umbelliprenin

Umbelliprenin (7-transfarnesyloxy coumarin) (UMB; C_2_H_30_O_3_; molecular weight = 366), a member of the 7-prenyloxycoumarins, is a naturally occurring compound belonging to the sesquiterpene class and isolated from *Ferula* species, including *Ferula szowitsiana* [42,43]. Recently, it was also isolated from *Artemisia absinthium* for the first time [44].

**Figure 1:**
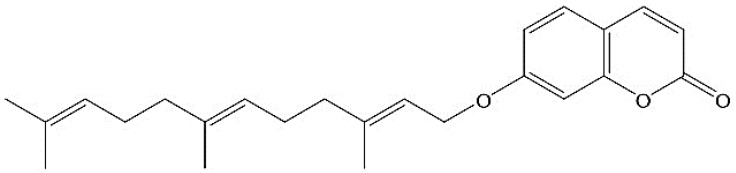
Chemical structure of Umbelliprenin (7-farnesyloxy coumarin).

The biological activities of coumarins are dependent on various types of substitutions in their structure [45]. The presence of an aryl group at Position 3 of a 4-hydroxycoumarin can contribute to inhibition of cell proliferation [46]. It has also been suggested that the proper substitution of a hydroxyl group at Positions 3 and/or 4 of coumarins is essential for designing effective cytotoxic agents [47]. Mousavi et al. showed that the cytotoxicity of UMB is due to the presence of a hydrophobic chain at the 1,2-benzopyrone ring C_7_OH position, but the main mechanism of action is still unknown. The presence of this hydrophobic chain increased the lipophilic property of the compound and therefore facilitated cell infiltration [48]. The molecular structure of UMB is depicted in Figure 1.

The effects of coumarins prenyl group on anticancer and anti-inflammatory activities have been studied in detail by Devji et al. [49]. They synthesized a series of prenylated and non-prenylated hydroxycoumarin derivatives and assessed their activities against human pancreatic PANC-1 cancer cells under deprived nutritional conditions. These findings showed that prenylated coumarins display higher cytotoxic activity against the cancer cells of PANC-1 relative to non-prenylated coumarins. It also revealed that the longer the prenyl side chain, the higher the cytotoxic activity. The introduction of a prenyl side chain into a molecule can increase its lipophilicity by enhancing access, affinity and interaction with the lipophilic membrane [50].

UMB can exhibit various pharmacological functions both in vitro [51,52,53] and in vivo [54,55], such as those related to antibacterial [42], antileishmanial [56], anti-inflammatory, antioxidant, anticancer, etc. [57]. Umbelliprenin’s inhibitory effects have been shown on 5-lipoxygenase [58] and on matrix metalloproteinases (MMPs) activities that may contribute to its anti-inflammatory actions [59]. In addition, its relaxant effect on smooth muscle has been revealed in the guinea pig model [60]. This review provides an updated compilation of the antineoplastic effects of UMB on various malignancies.

## 3. Role of Umbelliprenin (UMB) in Cancer and Its Molecular Targets

**Figure 2:**
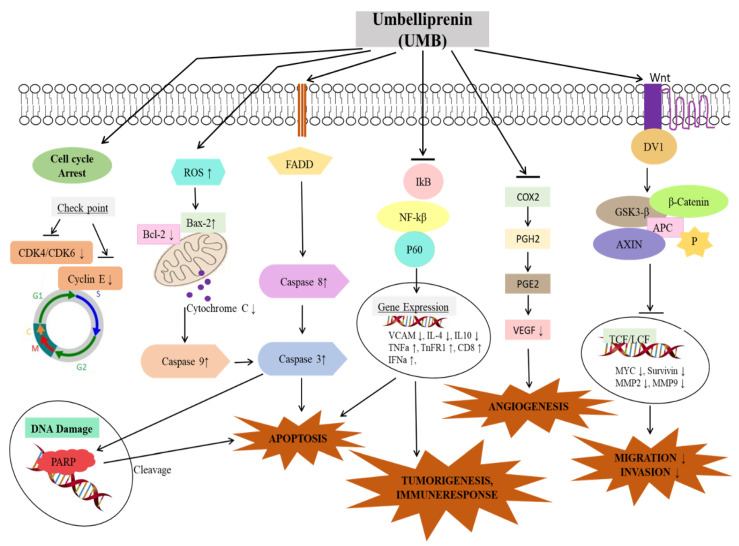
The various cell signaling cascades regulated by umbelliprenin (UMB). APC (Adenomatous polyposis coli), GSk-3-b (Glycogen synthase kinase 3 beta), COX2 (Cyclooxygenase-2), PGH2 (Prostaglandin H2), PGE2 (Prostaglandin E_2_), VEGF (Vascular endothelial growth factorNF-KB (Nuclear factor kappa B), VCAM (Vascular cell adhesion molecule), FADD (Fas-associated protein with death domain), CD8 (cluster of differentiation 8), ROS (Reactive oxygen species), CDK (Cyclin Dependent Kinase 4), P (Phosphorus).

Cancer results from the malfunctioning of multiple signaling cascades functional within the cells. These altered signaling cascades can contribute to the different important hallmarks of cancer [61]. However, diverse pharmacological compounds can be employed to inhibit the growth, metastasis and angiogenesis of tumor cells. Many natural compounds are favored to be used as cytotoxic agents against tumor cells due to their significant anti-cancer ability to target several oncogenic cascades and promote apoptosis [62,63,64]. UMB can exhibit cytotoxic effects against tumor cells with the ability to cause an inhibition in metastasis of tumor cells. UMB can affect multiple signaling cascades within the cells, i.e., intrinsic and extrinsic apoptotic pathways, Wnt and NF-ĸB activation, causes cell cycle arrest, as well as can modulate different inflammatory and immune related pathways (Figure 2) [44,45,46,47,48,49,50,51,52,53,54,55]. UMB plays a potential therapeutic role affecting different growth regulatory pathways as summarized in Table 1 and Table 2. The possible role of UMB in different type of cancers has been discussed below.

### 3.1. Breast Cancer

Breast cancer is a major clinical and health issue worldwide. It has a very high incidence and mortality rate in developing countries with inefficient prognosis, diagnosis, and treatment facilities [79,80,81,82]. Several reports over the past decades have indicated the cytotoxic potential of many important pharmacological agents against different types of breast cancers [83,84]. The studies on breast cancer cell line, i.e., MCF-7, showed increased cell death and decreased cell viability upon treatment with UMB in a dose dependent manner [51]. The IC50 values for UMB in different breast cancer cell lines varied in different studies ranging from 30–75 µM in a concentration dependent manner [48,65].

In vivo studies on breast tumor induced Balb/c mice indicated significantly reduced tumor growth and tumor volume upon the administration of UMB, which promoted apoptosis and cell cycle arrest to inhibit tumor growth. UMB exhibited significant anti-angiogenesis and anti-metastatic potential. It affected several oncogenic markers and down-regulated the levels of VCAM-1, MMP-9, MMP-2, NF-ĸB, CD31, COX-2, and VEGF. UMB also modulated immune responses by causing an up-regulation of IFN-γ and down-regulation of IL-4 [66]. However, detailed mechanistic studies are required to assess the mechanisms of its action on varying cell signaling pathways in breast cancer cells.

### 3.2. Lung Cancer

Lung cancer is the most common form of cancer across the globe with more death rate than any other cancer types [85,86,87,88,89]. Lung cancer can be divided into non-small cell lung cancer (NSCLC), with more than 80% incidence, and small cell lung cancer (SCLC), which is the more aggressive form of lung cancer [90,91]. Moreover, survival chances in metastasized lung cancer patients can be less than 5% [92]. The studies have shown antitumor potential of UMB on different types of lung cancer cell lines. A decreased cell viability in A549 (lung adenocarcinoma) cell lines was observed after treatment with UMB [67,68,69]. NSCLC and SCLC cell lines, i.e., A549 and QU-BD, respectively, exhibited reduced growth after the administration of UMB with IC50: 52 µM and 47 µM, respectively. MTT assay revealed a decrease in cellular viability while AnnexinV/PI staining exhibited increased apoptosis in lung cancer cells upon treatment with UMB [53].

In addition, multiple regulatory proteins can be affected upon treatment with UMB. A study suggested down-regulation of many important proteins, i.e., tubulin α-1B, HSP90, HSP27, hnRNP C1/C2, Cyclophilin, and tumor suppressor MST protein, and up-regulation of stathmin and calreticulin in lung cancer cell lines, i.e., QU-DB and A549 [70].

In vivo experiments in lung cancer-induced rats upon injection of UMB (2.5 mg/200 mL) intraperitoneally on alternate days showed increased cytotoxicity in lung cancer cells at IC50 of 51.6 ± 5.4 mM, with no adverse effects on normal spleen cells. UMB showed increased secretion of IL-4 and IL-10 in tumor-induced mice. Other important regulatory molecules, i.e., Foxp-3 and TGF-β, were also found to be down-regulated in lung cancer cells upon treatment with UMB [55].

### 3.3. Colon Cancer

Colon or colorectal cancer is the third most common cause of mortality after lung and breast cancer across the western world [72,93]. Studies using UMB as a potential antitumor agent (10 mg/mL) against colon cancer cells (DLD-1) showed reduced viability in a dose dependent manner [51]. IC50 values for UMB in human colon cancer cells (HT29) were noted to be 36.4 ± 1.6 and in mouse colon cancer cells (CT26) 51.4 ± 2.9 after 24 h of treatment [65]. UMB exhibited cytotoxic actions against invasive colon cancer cells (SW48), while non-invasive colon cancer cells (SW1116) showed enhanced proliferation when exposed to different concentrations of UMB [71]. The possible use of UMB in colon cancer therefore requires caution and detailed studies to assess the underlying mechanistic pathways.

The studies on colon cancer animal models showed a significant reduction in tumor size and angiogenesis after administration of UMB. The different molecular markers which were down-regulated by administration of UMB were VEGF, MMP-2, MMP-9, and CD31 while level of E-Cadherin was up-regulated. Ki-67 level was also down-regulated upon UMB treatment [94]. An induction of inflammatory and immune response by UMB was also observed by up-regulation of IFN-γ and down-regulation of IL-4 [78,95]. These findings warrant further investigation of UMB effects at cellular and molecular levels to decipher its detailed role against colon cancer.

### 3.4. Gastric Cancer

Gastric cancer is a lethal malignancy with significant mortality rate, and the patients generally develop resistance against chemotherapeutic agents employed for treatment [96,97,98,99,100]. In vitro studies on gastric cancer cell lines (AGS) showed that UMB can promote apoptosis in gastric cancer cells and reduce their viability, while it exhibited lesser cytotoxic effects on human gastric epithelial cell line (GES-1). In addition, detailed studies on gastric cancer cell lines (AGS) showed an increased expression of caspase-3, increased Bax/Bcl-2 ratio, and a decreased expression of MMP. This suggested the involvement of mitochondrial programmed cell death (PCD) pathways activated by UMB to promote apoptosis in gastric cancer cell lines. UMB also induced the cell cycle arrest at G0 and G1 phase of the cell cycle by altering the levels of proteins involved in cell cycle check points, i.e., Cyclin D, Cyclin E, CDK2, and CDK4 down-regulation and up-regulation of different CDK inhibitors [67]. In vivo studies on gastric cancer induced rat model (BGC-823 xenograft) showed inhibition of tumor in a dose dependent manner, i.e., 40.81% tumor suppression at 10 mg/kg and 63.64% tumor inhibition at 20 mg/kg [67].

A comparative analysis of UMB IC50 values against different gastric cancer lines indicated that it exhibited higher cytotoxic activity in AGS (IC50 11.74 μM) followed by BGC-823 gastric cancer cell line (IC50 24.62 μM), while least cytotoxic activity was observed in GES-1 normal gastric epithelial cells (IC50 97.55 μM). Wnt/β-catenin protein was observed to be activated at 30–50% in these tumor cell lines [72]. UMB treatment resulted in the down-regulation of WNT signaling pathway by reduction in the expression of phosphorylated GSK-3β and other effector proteins, i.e., β-catenin, as well as downstream oncogenic Survivin, c-myc, MMP2 and MMP9 proteins. Wnt antagonists may also work in a similar manner by inhibiting the formation of TCF-4/ β-catenin complex, which ultimately leads to the transcriptional down-regulation of c-myc and survivin [101]. UMB, on the other hand, showed dysregulation of Wnt signaling cascade by reducing the activity of TCF-4 reporter substantially [102]. Interestingly, no side effects of UMB were observed in gastric xenograft mouse model [73].

### 3.5. Prostate Cancer

Prostate cancer causes significant mortality among males worldwide [8]. Moreover, available therapeutic approaches, which include radiotherapy, chemotherapy, hormonal therapy, etc., have multiple limitations and there is need for new molecular targeted therapies to prevent the spread of this cancer [103,104]. UMB exerted substantial does dependent cytotoxic effects in prostate cancer cell lines (PC-3), while no cytotoxic effects were observed in normal cell line (HFF3) [51,67]. An over expression of 15-lipoxygenase-1 (LOX-1) can also serve as a potential biomarker for prostate cancer malignancy. LOX-1 regulates different metabolic pathways, which can aid prostate cancer cells to grow and become malignant. UMB showed inhibitory effects on LOX-1, thereby resulting in the attenuation of growth of PC-3 cells in a dose dependent manner. UMB also caused cell cycle arrest and induced apoptosis in prostate cancer cell lines [58]. Nevertheless, to understand the exact mechanism underlying these events, further studies are required.

### 3.6. Skin Cancer

Melanocytes are skin cells that contain melanin pigment. The cancer that develops in melanocytes is termed melanoma and it is usually localized to skin tissues, but some rare forms of melanoma are observed in intestines, eyes, and mouth as well [105]. Melanoma incidence has increased drastically among Caucasian populations as compared to other cancers in the past two decades. Malignant melanoma represents the most common form of fatal skin cancer [106,107,108,109]. UMB showed cytotoxic effects with IC50 of 12.3 mM in human malignant melanoma cell line (M4Beu). It acted in a dose-dependent manner and was found to be significantly cytotoxic to M4Beu cells, while no major cytotoxicity was observed in primary fibroblast cells. It reduced the serum-induced proliferation of M4Beu through cell cycle blockade at G1 phase and induction of dose-dependent apoptosis after 48 h of treatment [51]. In vivo assays using peroxynitrite and TPA as inducer of skin pappilomas indicated that UMB treated mouse showed significantly fewer pappilomas as compared to non-treated melanoma positive mouse in a time dependent manner [54].

### 3.7. Leukemia

Leukemia is the cancer of blood and can exist in different forms. In Western Europe, USA, and Canada, the most prevalent form of Leukemia is chronic Lymphocytic Leukemia (CLL), while in Asia, the frequency of CLL is comparatively lower [110]. CLL is a disorder of lymphocytes, which results in the accumulation of functionally disoriented cells [111]. The Jurkat cell line was incubated with different doses of UMB, i.e., 10, 20, and 40 µg/mL, and cytotoxicity was assessed at two different time points (24 and 48 h). UMB showed an IC50 value of 11.3 µg/mL, but the cytotoxicity was time- and dosage-dependent [74]. IL-4 is an inhibitor of apoptosis but treatment of Jurkat cells with 25 µM UMB induced apoptosis in Jurkat cells in the presence of IL-4 [52]. Another study showed a multi-step down-regulation of Mcl-1 in Jurkat cells treated with UMB [112]. Moreover, expression of Mcl-1 has a prognostic potential in CLL and the down-regulation of Mcl-1 by UMB can alter the balance of pro-apoptotic and anti-apoptotic proteins in cell thus promoting the mitochondrial regulated programmed cell death [75]. UMB also activated caspase dependent cell death mechanism in Jurkat cells. Elevated levels of capsase-8 and caspase-9 were observed after the treatment of Jurkat cells with UMB. Bcl-2 activity was also increased but in a time-dependent manner, and it decreased after 3 h of UMB treatment in Jurkat cells. No significant expression of Bax was observed in UMB treated Jurkat cells [76]. This suggests the activation of pro-apoptotic as well as anti-apoptotic proteins by UMB and the balance may be tilted towards the activation of pro-apoptotic proteins, which might explain the 36% apoptosis in those cells [52]. The combination of UMB with radiotherapy in MT-2 cells showed 89% viability at 20 µg/mL, while 63% of MT2 cells viability at 40 µg/mL concentration. This preludes the enhanced efficiency of radiotherapy in leukemic patients with co-administration of UMB with radiotherapy [77].

### 3.8. Fibrosarcoma

Morphologically dysfunctional fibroblasts with mesenchymal origin can lead to malignancy and tumor development such as fibrosarcoma [113,114]. This is a highly aggressive type of cancer with high resistance to chemotherapy and poor prognosis, which frequently leads to a high recurrence rate. The focus of treatment and therapy in fibrosarcoma had been on the induction of apoptosis sensitivity in tumor cells and inhibition of proliferation [115].

Matrix Metalloproteinase (MMPs) are calcium-dependent endopeptidases, which are responsible for the maintenance of extracellular matrix (ECM). This allows the tumor to invade into other tissues and organs and can promote metastasis. MMPs are the primary targets in the therapy development studies against fibrosarcoma [116]. In vitro studies showed that UMB displayed inhibitory effects on MMPs, which resulted in the inhibition of growth of fibrosarcoma-Wehi 164 cells even at lower concentrations. At 20 mg/mL of UMB, 70% inhibition of MMPs was observed, while the IC50 value of UMB cytotoxicity for fibrosarcoma-Wehi cells was 51 µg/mL. Zymographic analysis showed even lower IC50 for UMB (14 µg/mL) [59]. Thus, UMB has the potential to inhibit tumor metastasis and invasion into other tissues by inhibiting MMPs even at very low concentrations.

## 4. Strategies to Enhance UMB’s Bioavailability

UMB’s lower solubility in hydrophilic solvents can also affect its cytotoxicity in cell cultures as well as in vivo models. Low solubility of UMB reduces its bioavailability and hence it may not be adequately taken up by the cells. This requires the utilization of high concentrations of UMB to induce cytotoxicity in cells and achieve other desirable anti-cancer effects [117]. Interestingly, low bioavailability of UMB can be enhanced by using any drug carrier system. Encapsulation of UMB with nanoliposomes may augment its solubility in the cellular fluid and its bioavailability, which could significantly enhance its cytotoxic potential even at lower concentrations [117,118].

### 4.1. Nanoparticles Drug Delivery

In drug delivery, encapsulation of biologically active compounds with nanoparticles can serve as the ultimate tool in which nanoparticle encapsulation may function as a carrier of drug to the targeted region. This nano-encapsulation of biologically active compounds prevent the degradation of potentially therapeutic compounds and it can modify the compound’s solubilization in living system [119]. There are multiple drug delivery systems that provide an answer to the problems encountered in the targeted and efficient delivery of pharmacological agents. The important feature of any reliable drug delivery system is that it should be non-toxic in living systems, biodegradable, biocompatible, and, most importantly, it should be compatible with hydrophobic compounds. The drug delivery system should retain the compounds long enough to allow its sustained release in the living system with no cytotoxic effects of its own in the living system. Poly Lactic Acid (PLA) and Poly Lactic-Glycolic Acid (PLGA) polymers meet these criteria and can be used as efficient drug delivery systems [120,121]. In addition, another important mode of drug delivery is through the utilization of magnetic nanoparticles, which offers effective drug delivery options with minimum cytotoxicity of its own in the living system.

UMB-coated Magnetite (Fe_3_O_4_) nanoparticles have shown significant cytotoxic effects (IC50, 9 g/mL) against HT-1080 cell line. The proliferation of HT-1080 cells was remarkably reduced when UMB was used as UMB-Magnetite nano-formulation as compared to UMB alone. The cell viability in UMB-Magnetite treated cells was reduced to 45%, which supported the potential of this drug delivery system [122].

### 4.2. Liposomes

Multiple drug resistance (MDR) is a key mechanism used by tumor cells to overcome the cytotoxic effects of antitumor therapies. Tumor cells develop resistance by regulating the expression of different cell surface proteins, which effectively pumps the drug before or after entering the cells. An up-regulation of the *p-glycoprotein* gene in tumor cells is one such example of MDR [35]. Liposomes are nano-structured molecules, spherical in shape with two layers of layers of hydrophobic moieties serving as exterior covering and entrapped within this covering is hydrophilic/aqueous moiety [37,38]. Liposomes may bypass the drug resistance mechanism of tumor cells and have targeted delivery characteristics when used as drug delivery system. They can significantly enhance sustained release of drug at target sites with maximum cytotoxic concentration of drug still available [123,124,125].

MTT assay showed higher cell death and lower IC50 value of UMB in mouse mammary carcinoma cell lines when it was encapsulated in liposomes [118]. A comparative and concentration dependent study on A375 cell line (human melanoma cells) indicated that liposomal formulations of UMB showed cytotoxic effects in A375 cells even at lower concentrations at which DMSO dissolved UMB showed minimal toxicity [117].

## 5. Hormesis Effect Caused by UMB

Common dose-response curves show positive correlation between cytotoxicity of any drug with increase in the drug concentration. In some cases, reverse relation is observed, i.e., with increased drug concentrations, the cytotoxicity may be decreased. This phenomenon is termed hormesis. Over the past decades, the hermetic effects of several drugs have been widely studied [126]. There are different models to study hormesis. One model, which can analyze different concentration of drugs at a single time point, is called “Direct Stimulation”, while in the “Overcompensation Stimulation” model, multiple drug concentrations are studied at different time points [127].

The hormetic effects were observed with UMB in a dose-dependent single time point in direct stimulation study in Jurkat cells. UMB can activate intrinsic as well as extrinsic programmed cell death pathways by causing activation of caspase-8 and caspase-9, respectively [76,112]. Extrinsic pathways are ligand-receptor based pathways, which indicated that it might also modulate different receptors, which may alter the extent of apoptosis induced by the drug. However, hormetic effects of UMB require detailed studies and analysis of underlying pathways that may regulate its diverse pharmacological activities [127].

## 6. Conclusions and Future Perspectives

This review provides a comprehensive overview about the diverse anticancer potential of UMB, a sesquiterpene predominantly isolated from the Ferula species. The effects of UMB may be primarily exerted through the induction of extrinsic and intrinsic apoptosis, cell cycle arrest and the inhibition of cell proliferation along with modulation of multiple cell signaling mechanisms in breast, lung, colorectal, gastric, prostate, melanoma, leukemia, and fibrosarcoma. Amidst various signaling pathways, Wnt and NF-κB were found to be the most significantly affected by UMB in different cancers. Therefore, UMB can be used as a potent anticancer agent against several malignancies that have developed resistance to chemotherapy. However, its effective concentrations against diverse tumor cells may vary depending on the type of cell and in vivo model system used. Hence, detailed preclinical studies are required to assess the pharmacokinetic properties of UMB to facilitate its rapid application in cancer patients. This may open new perspectives for the development of natural compounds such as UMB as therapeutic agents for cancer treatment.

## Figures and Tables

**Table 1:** In vitro anticancer actions of UMB.

Cancer	Cell Line	Dose/IC 50	Biological Effects	Molecular Targets	Reference
Breast cancer	MCF-7 4T1 mammary carcinoma cells	73.4 µM,30.6 µg/mL, 59.7 µM 30.6–62.2 µg/mL for 24, 48, 72 h	Mitochondrial Apoptosis, rounding of cell, reduction of cell volume, DNA fragmentation, cell cycle arrest at G1 phase, Antiproliferative	↑Bax	[48,51,65,66]
Lung cancer	M4Beu, A549, QU-DB	52 µM, 133 µM 47 µM	Apoptosis, anti-proliferation, Anti-angiogenesis, Inhibit migration, Lymphocyte proliferation	↓HSP90, ↓HSP27, ↓GRP94, ↓vimentin, ↓hnRNP C1/C2, ↓p97/VCP, ↓NDUFS3, ↓importin-α2, ↓importin-β1, ↓tubulin α-1B, ↓FKBP4, ↓SF3a3, ↓cyclophilin B, ↓APRT, ↓DDAH-2, ↓VHR, ↓annexin A4, ↓prohibitin, ↓proteasome α-1, ↓MST, ↓keratin-1, ↓heat shock protein 90 kDa, ↑Nipsnap1, ↑glycine-tRNA ligase, ↑stathmin (tumorigenic), ↑calreticulin	[51,53,67,68,69,70]
Colon cancer	DLD1, HT29 human CT26 mouse colon carcinoma SW48 cells SW1116 cells	36.4 µg/mL 51.4 µg/mL 69 µM for 72 h	Reduced cell viability, Apoptosis	−	[51,65,71]
Gastric cancer	AGS, BGC-823, GES-1	11.74 µM, 24.62 µM	Mitochondrial apoptosis, ROS generation, increased, cycle arrest at G0/G1 phase,	↑Bax, ↑caspase 3 expression, ↑p27, ↑P16, ↑Rb 4, ↓MMP9, ↓MMP2, ↓cyclin D, ↓cyclin E, ↓CDK4, ↓CDK2, ↓Wnt, ↓β-catenin, ↓Survivin, ↓c-myc, activation of PARP cleavage	[67,72,73]
Prostate cancer	PC3, DU145	4.3 µg/mL	Reduced cell viability, Apoptosis, DNA damage, cell cycle arrest at G1 phase,	↓15-LOX expression	[51,58]
Skin cancer	SK-MEL-28 M4Beu	58.1 µM 25 mM	Apoptosis, Antiproliferative, cell cycle arrest at G1 phase,	−	[68]
Leukemia	Jurkat T-CLL Raji B-CLL, MT2 cells, ATLL cell line	11.3 µg/mL, 25 µM, 40 µg/mL + radiotherapy	Apoptosis, decrease cell viability	↓Mcl-1 level, ↑caspase 3, caspase 9, caspase 8, ↑Bcl2	[52,74,75,76,77]
Fibrosarcoma	Wehi 164 cells	14 µg/mL	Apoptosis	↓MMP	[59]
Esophageal carcinoma	KYSE-30	24.32 µg/mL	Reduced cell viability	−	[74]
Cervical cancer	HeLa	20.22 µg/mL	Reduced cell viability	−	[69,74]
Ovarian cancer	CH1, A2780	37.2 µg/mL	Reduced cell viability	−	[51,68,69]
Glioma	GL26 mouse cancer cells A172 human cancer cells	46.1 µg/mL 37.9 µg/mL For 48 h	Reduced cell viability	−	[65]

− shows not determined, ↓ shows down-regulation, ↑ shows up-regulation.

**Table 2:** In vivo anticancer effects of UMB.

Cancer	Model		Phenotypic Effect	Molecular Target	References
Breast Cancer	4T1 tumor-bearing Balb/c mice	200 µL of UMB (12.5 mg/mL)	Inhibit tumor growth, apoptosis, cell cycle arrest, anti-angiogenesis, ant-metastasis, anti-inflammatory, Th1 response	↓VEGF, ↓COX2, ↓MMP2, ↓MMP9, NF-κB, ↓CD31, ↓CD31, ↓VCAM1, ↓IL-4 cytokines, ↑IFNγ ↑E-cadherin, ↑TNFR1	
Lung cancer	LLC cells induction in mice	2.5 mg/200 mL	Apoptosis, augmentation, cell mediated immune response	↓IL-4, ↓IL-10, ↑TNF-a, ↑IFN-g, ↓Foxp3, ↓TGF-b	[55]
Colon cancer	CT26 cell line injected in Mice		Apoptosis, reduction of tumor size, angiogenesis, antiproliferative anti-metastasis, immune response	↓VEGF, ↓MMP2, ↓MMP9, ↓CD31, ↓Ki-67, ↓MMP2, ↓MMP9, ↓IL-4 decrease, ↑E-cadherin, ↑IFN-γ	[78]
Gastric cancer	human gastric cancer BGC-823 xenograft models	43.33% (20 mg/kg)	Reduction in tumor volume, decrease in rats body weight	−	[67]
Skin cancer	vivo two-stage mouse skin		Less papillomas	−	

− shows not determined, ↓ shows down-regulation, ↑ shows up-regulation.

## Abbreviations

UMB	Umbelliprenin
BAX	Bcl2-Associated X Protein
BCL-2	B-Cell Lymphoma-2
CDK	Cyclin Dependent Kinase
CRC	Colorectal Cancer
CRPC	Castration Resistance Prostate Cancer
EGFR	Epidermal Cell Growth Factor Receptor
EMT	Epithelial-Mesenchymal
IL-4	Interleukin 4
IL-10	Interleukin-10
MMP-9	Matrix Metalloproteinase 9
MMP-2	Matrix Metalloproteinase 2
NF-kB	Nuclear Factor Kappa Light Chain Enhancer of Activated B Cells
PARP	Poly ADP-Ribose Polymerase
VEGF	Vascular Endothelial Growth Factor
MDR	Multiple drug resistance
PLGA	Poly Lactic-Glycolic Acid
PLA	Poly Lactic Acid
TNF	Tumor necrosis factor
Foxp	Forkhead box protein
IFN	Interferone
15-LOX	Lysyl oxidase
TGF	Transforming Growth Factor
µg	Microgram
µM	Micromole
CLL	chronic Lymphocytic Leukemia
Rb-4	Retinoblastoma 4
VHR	vaccinia H1-related
DDAH	dimethylarginine dimethylaminohydrolase
APRT	Adenine Phosphoribosyltransferase
MST	Macrophage-stimulating protein
SF3A3	Splicing factor 3A subunit 3
